# Impacts and Policy Implications of Metals Effluent Discharge into Rivers within Industrial Zones: A Sub-Saharan Perspective from Ethiopia

**DOI:** 10.1007/s00267-017-0970-9

**Published:** 2017-12-09

**Authors:** E. Zinabu, P. Kelderman, J. van der Kwast, K. Irvine

**Affiliations:** 1 Wollo University, P.O. Box 1145, Dessie, Ethiopia; 2 IHE Delft Institute for Water Education, P.O. Box 3015, 2601 DA Delft, Netherlands; 3 Aquatic Ecology and Water Quality Management, Wageningen University, P.O. Box 47, 6700 AA Wageningen, Netherlands

**Keywords:** Ethiopia, metals, Industrial effluents, Kombolcha, Policy enforcement

## Abstract

Kombolcha, a city in Ethiopia, exemplifies the challenges and problems of the sub-Saharan countries where industrialization is growing fast but monitoring resources are poor and information on pollution unknown. This study monitored metals Cr, Cu, Zn, and Pb concentrations in five factories’ effluents, and in the effluent mixing zones of two rivers receiving discharges during the rainy seasons of 2013 and 2014. The results indicate that median concentrations of Cr in the tannery effluents and Zn in the steel processing effluents were as high as 26,600 and 155,750 µg/L, respectively, much exceeding both the USEPA and Ethiopian emission guidelines. Cu concentrations were low in all effluents. Pb concentrations were high in the tannery effluent, but did not exceed emission guidelines. As expected, no metal emission guidelines were exceeded for the brewery, textile and meat processing effluents. Median Cr and Zn concentrations in the Leyole river in the effluent mixing zones downstream of the tannery and steel processing plant increased by factors of 52 (2660 compared with 51 µg Cr/L) and 5 (520 compared with 110 µg Zn/L), respectively, compared with stations further upstream. This poses substantial ecological risks downstream. Comparison with emission guidelines indicates poor environmental management by industries and regulating institutions. Despite appropriate legislation, no clear measures have yet been taken to control industrial discharges, with apparent mismatch between environmental enforcement and investment policies. Effluent management, treatment technologies and operational capacity of environmental institutions were identified as key improvement areas to adopt progressive sustainable development.

## Introduction

In many sub-Saharan countries, water pollution is an ongoing and acute challenge for sustainable development (Alcamo et al. 2012; Hove et al. 2013). Environmental regulatory structures may be in place, but pressures to attract investors for industrial activities may reduce regard for pollution abatement (Bertinelli et al. 2012; Sikder et al. 2013; Xu et al. 2014). Policies to promote economic gains can lead to a path of “pollute now; clean-up later” (Alcamo et al. 2012; Rudi et al. 2012; Sikder et al. 2013). The seemingly existing paradox of crafting good environmental policies but low enforcement has a risk of making the industrial growth unsustainable. Also, many industrial technologies are quite old and there is a tendency to import cheaper technologies to cope with environmental requirements under increasing pressure of economical returns (Bertinelli et al. 2006; Rudi et al. 2012). According to the environmental Kuznets curve (Grossman and Krueger 1991), the ratio of socio-economic development to pollution may increase till the technology reaches the scrapping age, when operational cost can no longer cover market value for environmental quality (Bertinelli et al. 2012). Thereafter, this ratio will decrease only if improvement of the technologies reduce environmental impact.

Industrial effluents containing metals, and their accumulation in sediments and biota, present a persistent threat to ecosystems health (Gaur et al. 2005; Jining and Yi 2009; Kelderman 2012; Xu et al. 2014). This holds also for sub-Saharan African countries, where regular monitoring is limited (Akele et al. 2016; Ndimele et al. 2017). Thus, identifying effluent concentrations and discharge management are of increasing importance if environmental risks and hazards are to be addressed (Rudi et al. 2012).

**Fig. 1:**
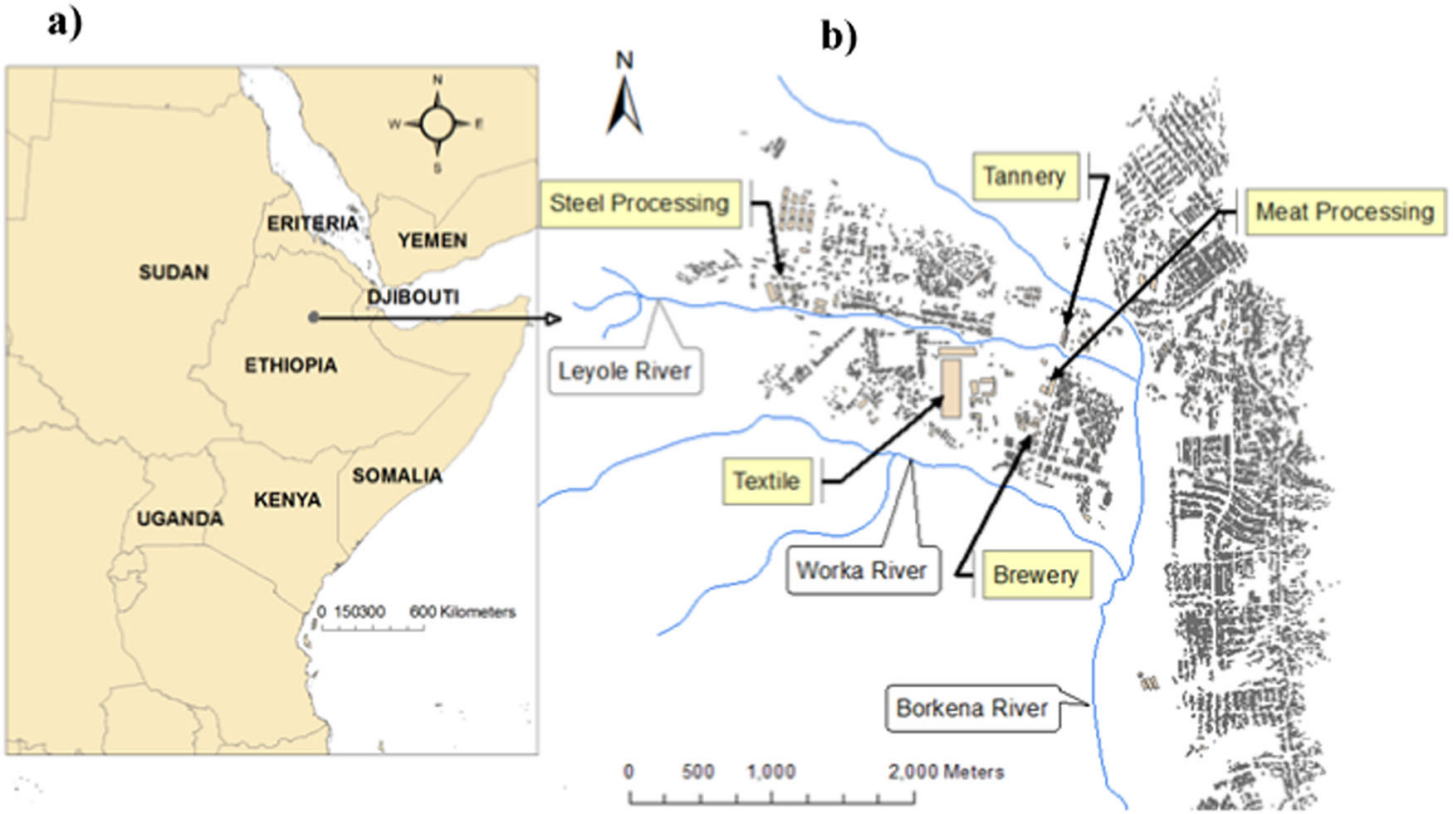
Location of the study area. **a** Study area in East Africa, northern Ethiopia, **b** Kombolcha industrial area (source: Kombolcha administration city office (2014)

This study is focused on the industrial city of Kombolcha in Ethiopia (Fig. 1), a typical sub-Saharan African city where urbanization and industrialization are growing fast but monitoring resources are poor. Data for industrial effluents and water quality are scant here and the threat to sustainable development is unknown. Backed by the government, the city’s industries are growing fast, with the expansion of existing ones and ambitions to attract foreign investors for new ones. These industries discharge effluents into nearby Waterways. While industrial pollution control policies have been formulated for the country, the environmental institutions at regional and local levels are yet to be evaluated with respect to their role for sustainable industrial development. In this study, we examined the dissolved metals: chromium (Cr), zinc (Zn), copper (Cu) and lead (Pb) in the effluents of five industries. The study aimed to (1) quantify the metal concentrations and loadings from these industrial effluents; (2) assess metal concentrations in the effluent mixing zones of the receiving rivers; and (3) evaluate the industries compliance with water quality guidelines, and identifying gaps in pollution control to recommend policy options.

## Materials and Methods

### Study Area

**Fig. 2:**
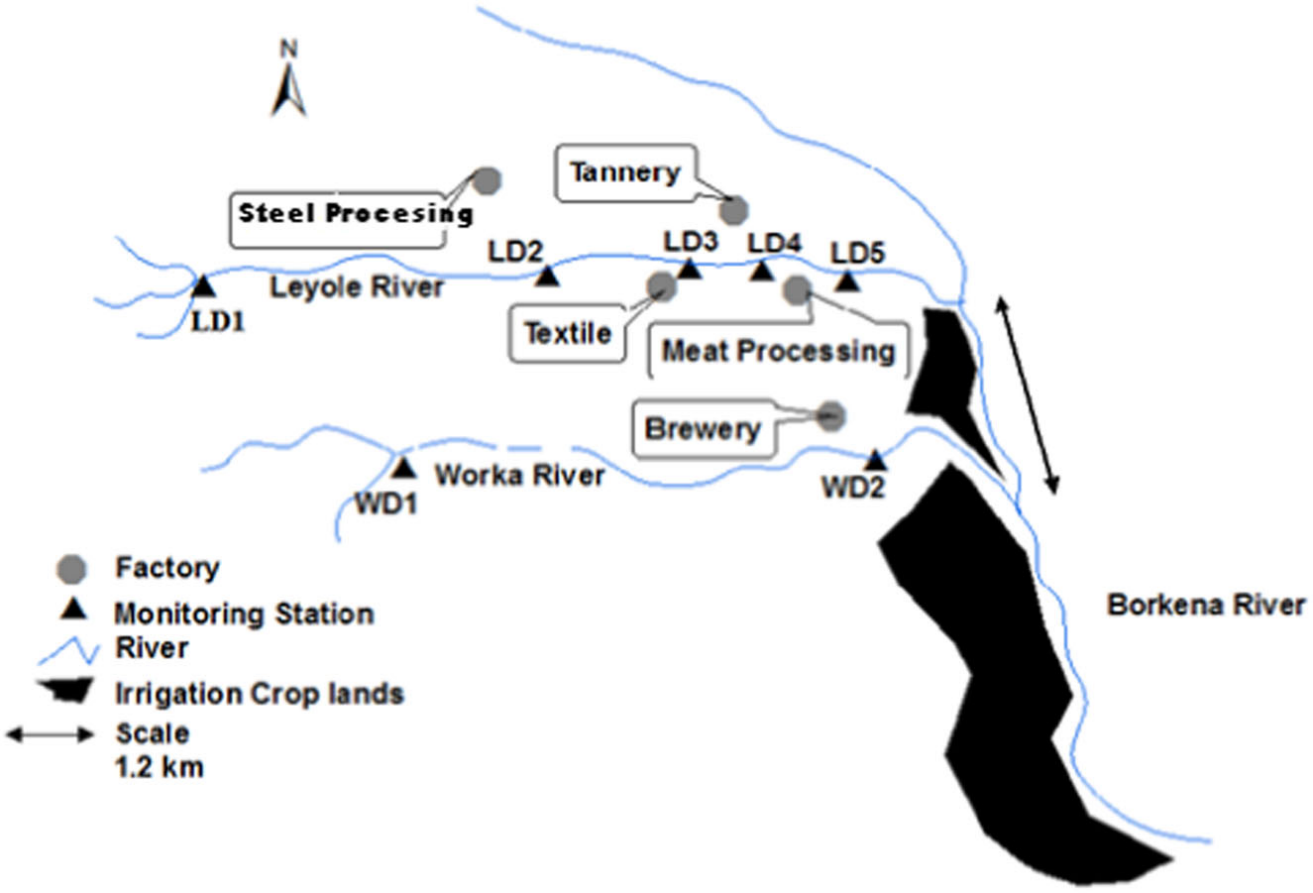
Schematic outlines of the rivers receiving the effluents of five industries, the factories’ effluent discharge points and the monitoring stations and codes (LD1 (Confluence point of upper part tributaries and start of upstream Leyole river); LD2 (Steel processing effluent mixing zone in the Leyole river); LD3 (Textile effluent mixing zone in the Leyole river); LD4 (Tannery effluent mixing zone in the Leyole river); LD5 (Meat processing effluent mixing zone in the Leyole river); WD1 (Upstream Worka river); and WD2 (Brewery effluent mixing zone in the Worka river)) along the Leyole and Worka rivers flowing into the Borkena river

Kombolcha, in the North central part of Ethiopia, covers 125 km^2^ (Fig. 1a), comprising rural upland landscapes in the north and populated lowlands in the south. Different land use types exist in the area, with extensive agriculture and forest land in the upland zone, and peri-urban and heavily urbanized and industrial areas mainly in lowland plains. The soils of the study area are generally vertisol while the river banks and the foot of upstream hills are dominated by Fluvisols and Cambisol soil types, respectively (Zinabu 2011). The area has annual bimodal rainfall seasons, usually from February to April, with heavier rainfall from July to September. Several tributary rivers rise from the surrounding escarpments and drain into two rivers, the Leyole and Worka rivers, which flow through an industrial zone of Kombolcha (Fig. 1b). The Leyole river receives effluents from the following four factories (Fig. 2):

• Steel processing factory, producing 26,000 tons per year of corrugated iron sheet;
• Textile factory, producing 22 million textiles per year, in garment production and dyeing;
• Tannery (not operating in 2013), soaking 1000 sheep skins and 3200 goat skins per day;
• Meat processing factory, dressing maximally 200 cattle per day.

The Worka river receives effluents from a brewery factory, with a production capacity of 250,000 bottles of beer per day (330 mL beer per bottle).

### Sample Collection, Preservation and Analysis

#### Factories effluent and river water sampling

Sampling was done in two bimonthly (15/30) monitoring campaigns during the rainy season from June–September in 2013 (campaign C1) and 2014 (C2). Samples were taken to measure total dissolved Cr, Cu, Zn, and Pb directly in the five factories effluents. Additional monitoring took place in the effluents mixing zones of the Leyole and Worka rivers (LD2-5; WD2; Fig. 2). Stations at the confluence of three tributaries in the upper part of the Leyole river (LD1) and confluence of two tributaries in the upper part of the Worka river (WD1) were located upstream of the industrial zone. The latter provides a theoretical baseline for estimates of pollution from industrial effluents. Results were used in evaluating river water quality.

pH and EC (electrical conductivity) were measured in situ using a portable pH (WTW, pH340i) and EC (WTW, cond330i) meter, respectively. The industrial effluent samples were taken directly from the discharge pipes using a 100 mL polyethylene (PE) sample container. Grab samples were taken based on equally spaced time intervals or volume, and were then mixed to make a composite sample. The choice for either equal time or equal volume sub-samples was based on the way the effluents were discharged by the factories. For factories with intermittent batch discharge of process effluents, three grab samples were collected at the beginning, halfway through, and at the end of the discharge of the effluent. For factories with continuous effluent discharges, eight grab samples were taken at equally spaced time intervals (i.e. every 3 h in a 24 h period), and samples were mixed in equal batches. In total, 40 (8*5) effluent samples have been taken in both 2013 and 2014. Water samples were also taken in the effluents mixing zones within a 5 m long section immediately downstream of the effluent discharge points into the Leyole and Worka rivers. As the mixing zones of Kombolcha’s factories were not exactly determined, we assumed that a 5 m long section was sufficient for complete mixing of the effluent, containing both the zone of initial dilution (ZID), near the effluent outfall, and the chronic mixing zone (impact zone) (Schnurbusch 2000; Alonso et al. 2016). The samples were collected at 1/4, 1/2, and 3/4 of the width of the river, and a composite sample prepared from equal volume proportions in a 100 mL PE container. Thus eight river water samples were taken for the two monitoring campaigns at the seven stations (Fig. 2), yielding a total of 56 samples in both 2013 and 2014. Both the river water and effluent samples were preserved with 1 mL concentrated H_2_SO_4_ to keep the pH < 3 in order to prevent metal adsorption onto the PE container wall (Rice et al. 2012). Within 15 to 135 days, samples were air-transported to the IHE Delft laboratory, located in Delft, the Netherlands and kept in a cold room (<4 °C). Following the ISO 5643-3 guideline, the samples preservation time was always <6 months (ISO 2003). A 10 mL sub-sample of the effluent was then filtered over a Whatman GF-C glass microfiber filter (pore size 1.25 µm) and diluted with Milli-Q water. The metals concentrations were measured using ICP-MS (Inductively Coupled Plasma Mass Spectrometry), XSERIES 2 IUS-MS. All analyses were done in accordance with APHA-AWWA-WPCF-2012 (Rice et al. 2012).

### Hydrology Measurements

The industrial effluent discharges were measured while collecting the above water samples, using the volumetric method, a simple and accurate method for very small flows with free-fall, such as at the outfall of a pipe or culvert (Hamilton 2008). The time to fill a known volume (40 L) of effluent container was first estimated for each factory’s effluent discharge pipe and flow rates were calculated by dividing the volume by the time to fill the container.

In order to estimate the dilution capacities of the Leyole and Worka rivers, daily flow depths of the river water were recorded twice a day during the sampling campaigns for four months, from 1 July to 30 September 2013 and 2014, in line with Herschy (1985). The measurements were taken at LD1, LD5 and WD2 (Fig. 2). In addition, 12 discharges were measured in three flow regimes (low, medium and high flows) following the methods outlined in ISO regulation 1100-2 (Voien 1998). The river channel cross-section was first divided into vertical subsections. In each subsection, the area was estimated by measuring the width and depth of the subsection, and the water velocity was then determined using a current meter (Price-Type AA) or a pigmy-current meter. For low flows and shallow water depths at the start (i.e. in June) of the campaigns, a pigmy meter was used, whereas a vertical axis cup current meter was used for medium to high flows. The discharge (m^3^/sec) in each subsection was computed by multiplying the subsection area by the measured velocity, and the total discharge estimated by summing up the discharges for each subsection. Stage-discharge rating curves were then prepared following Kennedy (1984). The least mean square method was used to estimate rating curve coefficients and, from that, the flow rates of the Leyole and Worka rivers (Das 2014).

### Statistical Techniques

All water quality data analyses were performed in R statistical packages (R Core Team 2015). Normality of the data was first tested using a Shapiro-Wilk normality test (Shapiro and Wilk 1965; Degens and Donohue 2002), in order to choose the required statistical methods for further data analysis. Descriptive statistics were carried out for the results of the sample analyses. Here the data set for each station was found to be asymmetrically distributed with the mean values affected by a few high or low values (Tables 1, 2). To best summarize these data sets, median values were selected for better representation of central tendency concentrations at each station (Bartley et al. 2012). These median values were compared with environmental guidelines.

**Table 1:** Estimates of EC, pH, and of effluent concentrations and guidelines (μg/L), as well as standard errors (μg/L), effluent discharges (L/s) and daily loadings (g/day) of metals in the five factories’ effluents, during the first (C1) and second campaign (C2), from June–September 2013 and 2014, respectively. For the effluent loadings, the “direct median loading method” was used, *n* = 8

Factory		Steel		Textile		Tannery		Meat processing		Brewery	
Campaign (*n* = 8)		C1	C2	C1	C2	C1	C2	C1	C2	C1	C2
EC (µS/cm)	Median	5730	3800	932	760	710	4470	1480	1590	920	1130
	Mean	14,400	4000	920	800	2200	5200	920	1200	2100	1800
	Maximum	78,000	7460	1190	1010	10,570	12,280	1170	1740	7100	3070
	Minimum	1430	620	730	480	450	800	560	740	720	1,070
	Standard error	920	790	54	63	1240	1500	77	116	731	247
pH	Median	6.1	5.5	10.3	8.2	7.8	7.4	8.2	7.2	11.1	11.2
	Maximum	6.1	10.9	10.2	8.8	7.8	8.1	8.2	8.2	11.8	11.4
	Minimum	0.4	2.2	7.5	7.7	7.4	5.6	6.7	7.1	5.2	6.9
	Standard error	0.7	1.1	0.4	0.1	0.0	0.4	0.4	0.1	0.7	1.1
Cr	Median (µg/L)	89	17	4.1	3.1	6.1	26,800	2.2	9	10	40
	Mean (µg/L)	150	32	4.1	45	22	33,270	2.1	60	8	36
	Maximum (µg/L)	485	85	4.9	297	131	64,600	2.1	215	16	77
	Minimum (µg/L)	2.1	1.1	2.2	2.1	2.3	813	2.3	1.1	2.1	2.9
	Standard error (µg/L)	60	11	0.7	36	17	7,850	0	34	2	8
	USEPA guideline^a^ (µg/L)	1300	1300	N.A.^b^	N.A.	12,000	12,000	N.A.	N.A.	N.A.	N.A.
	EMoI guideline^c^ (µg/L)	1000	1000	1000	1000	2000	2000	N.A.	N.A.	N.A.	N.A.
	Mean effluent (L/s)	1.7	2.2	15.4	16.5	6.8	8.4	11	8.8	8.2	21
	Loadings (g/day)	11	4	3	4	2.5	18,500	1.1	6	4	40
Cu	Median (µg/L)	65.2	99	14	6.9	11	15	9.1	3.1	25	26
	Mean (µg/L)	125	137	58	13	125	22	31	6.8	111	43
	Maximum (µg/L)	440	340	290	50	290	85	160	20	290	200
	Minimum (µg/L)	8.5	0.1	3.5	0.1	8.1	0.1	2.5	0.1	4.9	1.4
	Standard error (µg/L)	45	54	34	6	51	0	10	20	3	47
	USEPA guideline (µg/L)	1300	1300	N.A.	N.A.	N.A.	N.A.	N.A.	N.A.	N.A.	N.A.
	EMoI guideline (µg/L)	2000	2000	2000	2000	N.A.	N.A.	N.A.	N.A.	N.A.	N.A.
	Mean effluent (L/s)	1.7	2.2	15.4	16.5	6.8	8.4	11	8.8	8.2	21
	Loadings (g/day)	6	20	22	9	6.3	10	5	3	17	29
Zn	Median (µg/L)	60,040	155,750	120	110	90	280	110	140	150	210
	Mean (µg/L)	170,000	172,600	200	230	980	390	160	150	210	220
	Maximum (µg/L)	662,700	450,700	7190	640	7190	1250	180	330	720	440
	Minimum (µg/L)	14,100	14,150	26	29	26	130	25	44	20	68
	Standard error (µg/L)	87,800	50,110	76	85	887	0	125	43	33	76
	USEPA guideline (µg/L)	3500	3500	N.A.	N.A.	N.A.	N.A.	N.A.	N.A.	N.A.	N.A.
	EMoI guideline (µg/L)	5,000	5,000	5,000	5,000	N.A.	N.A.	N.A.	N.A.	N.A.	N.A.
	Mean effluent (L/s)	1.7	2.2	15.4	16.5	6.8	8.4	11	8.8	8.2	21
	Loadings (g/day)	4950	17,300	207	160	54	210	47	100	114	280
Pb	Median (µg/L)	5.1	8.2	2.9	1.1	2.1	2.1	2.9	1.1	5.9	1.1
	Mean (µg/L)	16	22	4.1	1.7	3.1	130	3.2	2.1	4.9	2.1
	Maximum (µg/L)	43	66	7.1	4.1	3.9	1670	4.1	2.9	8.1	2.9
	Minimum (µg/L)	2.1	0.6	2.1	0.6	2.1	0.6	2.1	0.6	2.1	0.6
	Standard error (µg/L)	5.7	9.5	0.7	0.7	0.3	0.0	233	0.2	0.2	0.7
	USEPA guideline (µg/L)	120	120	N.A.	N.A.	N.A.	N.A.	N.A.	N.A.	N.A.	N.A.
	EMoI guideline (µg/L)	500	500	500	500	N.A.	N.A.	N.A.	N.A.	N.A.	N.A.
	Mean effluent (L/s)	1.7	2.2	15.4	16.5	6.8	8.4	11	8.8	8.2	21
	Loadings (g/day)	1	1.3	3	1	1	4	1	0.6	3	2

^a^ USEPA (2014)

^b^ N.A. not available; no guideline concentration is given

^c^ EMoI (2014)

**Table 2:** Estimates of EC, pH, and the metal concentrations (μg/L), flow rates (L/s) and loadings (g/day) for the industrial effluents mixing zones (M.z.) of the Leyole and Worka rivers. The flow rates (in italic) at LD 2–4 were estimated by interpolation, taking the average of flow rates at LD1 and LD5. The loadings were calculated as the product of median concentrations and flow rates of the rivers

Station		LD1		LD2 (M.z. steel)		LD3 (M.z. textile)		LD4 (M.z. tannery)		LD5 (M.z. meat proc.)		WD1		WD2 (M.z. Brewery)	
Campaigns (*n* = 8)		C1	C2	C1	C2	C1	C2	C1	C2	C1	C2	C1	C2	C1	C2
EC (µS/cm)	Median	620	530	570	460	750	550	750	980	760	850	430	340	680	1240
	Mean	540	490	540	420	700	550	740	1050	770	850	400	350	700	1280
	Maximum	718	685	617	574	1080	650	1010	1480	1110	1260	480	470	990	2850
	Minimum	200	150	280	180	520	400	420	710	440	290	290	240	430	570
	Standard error	66	65	38	43	62	32	66	105	69	113	24	27	71	241
pH	Median	7.5	8.0	8.1	8.3	8.3	8.1	7.8	7.9	7.6	7.6	8.1	8.4	6.3	9.5
	Maximum	8.3	8.2	8.5	8.7	8.8	8.5	8.2	7.9	8.5	7.9	8.5	8.7	9.5	11.2
	Minimum	7.3	7.2	7.2	7.6	7.9	7.6	7.1	7.4	7.4	7.3	6.4	8.0	4.4	6.9
	Standard error	0.8	0.13	0.8	0.13	0.89	0.13	0.83	0.07	0.84	0.09	0.83	0.1	0.74	0.58
Cr	Median (µg/L)	3.9	2.1	12	6.1	7.9	51	9.1	2660	8.9	280	2.1	2.1	7.1	38
	Mean (µg/L)	3	440	11	380	6.9	230	9	6880	11	4280	3.1	37	7.9	30
	Maximum (µg/L)	21	2690	44	2160	25	1130	15	25,900	16	18,250	4.9	154	13	73
	Minimum (µg/L)	1.9	1.1	2.1	0.7	2.1	0.7	1.9	206	2.1	26	2.1	1.2	2.1	2.1
	Standard error (µg/L)	4.1	330	5.1	260	3.1	140	8.9	3360	6.1	2580	0.1	22	1.1	9.1
	Mean river flows (L/s)	98	184	*120*	*240*	*135*	*277*	*138*	*287*	142	296	360	1320	360	1,320
	Loadings (g/day)	34	32	*124*	*124*	*93*	*1220*	*110*	*66,000*	*110*	*7260*	*62*	*228*	*218*	*4330*
Cu	Median (µg/L)	23	0.4	17	14	63	41	10	21	14	27	8	0.2	13	33
	Mean (µg/L)	80	300	83	270	100	160	41	85	65	190	51	34	73	350
	Maximum (µg/L)	303	1900	248	1540	250	830	250	360	270	1180	270	150	270	2450
	Minimum (µg/L)	3.1	0.1	6.9	0.1	4.1	0.1	2.9	0.1	3.1	0.1	2.1	0.1	3.1	0.1
	Standard error (µg/L)	37	240	36	190	37	100	30	45	33	140	33	22	35	300
	Mean river flows (L/s)	98	180	*120*	*240*	*130*	*280*	*140*	*290*	140	300	360	1,320	360	1,320
	Loadings (g/day)	195	6	*176*	*290*	*735*	*980*	*119*	*521*	*172*	*691*	249	23	*404*	*3,760*
Zn	Median (µg/L)	72	110	95	520	71	187	30	205	81	214	41	137	106	194
	Mean (µg/L)	77	110	109	886	91	525	52	384	127	528	67	151	194	175
	Maximum (µg/L)	126	3310	367	2780	218	1600	131	1050	611	2120	143	338	855	278
	Minimum (µg/L)	26	16	29	9.1	54	34	15	67	15	25	8.9	12	14	46
	Standard error (µg/L)	15	402	37	365	21	209	17	127	65	250	19	45	92	29
	Mean river flows (L/s)	98	184	*120*	*240*	*135*	*277*	*138*	*287*	142	296	360	1320	360	1320
	Loadings (g/day)	610	1750	*985*	*10,800*	*828*	*4480*	*358*	*5080*	*994*	*5470*	1280	15,630	*3300*	*22,130*
Pb	Median (µg/L)	2.1	1.1	2.9	1.1	2.9	3.1	3.9	5.1	3.1	0.8	2.1	1.1	3.9	1.1
	Mean (µg/L)	1.1	11	1.1	9.9	1.1	8.1	0.4	128	1.1	7.9	3.1	2.1	2.1	1.1
	Maximum (µg/L)	4.9	70	6.1	60	4.9	34	4.1	980	4.1	44	3.9	7.1	4.9	5.1
	Minimum (µg/L)	2.1	1.1	2.1	1.1	1.9	1.1	2.1	0.6	2.1	0.6	2.1	1.1	2.1	1.1
	Standard error (µg/L)	0.4	8	0.7	7	0.4	3.9	4.1	121	0.4	5.1	0.3	0.7	0.2	0.4
	Mean river flows (L/s)	98	184	*120*	*240*	*135*	*277*	*138*	*287*	142	296	360	1320	360	1320
	Loadings (g/day)	17	16	*31*	*21*	*35*	*72*	*48*	*124*	*37*	*20*	62	114	*124*	*114*

### Metals Mass Transport Loadings

The loading (g/day) estimations were computed in a Flux 32 software environment, an interactive computer programme used to estimate the loadings of water quality constituents such as nutrients, metals and suspended sediments. The software incorporates six methods of estimating loadings of water quality constituents (Walker 1987, 1990). As loadings by the factory effluents are not expected to vary much with effluents flows. A “direct loading median” method was used by determining medians of the loadings of a metal at each sampling time. These were derived from median of the product of metal concentrations and effluent of the factories during each sampling. The method is somewhat different from “numeric integration” which is based on the average of the loadings at each sampling time (Walker 1987). Similarly, the loadings in the effluent mixing zones of the rivers were estimated using the product of median concentrations of the metals and average flows of the river at a station. This method is appropriate for cases in which concentrations of metals tend to be inversely related to flows, and loadings do not vary with river flow (Walker 1987). This often occurs at effluent mixing zones for industries, as the flow and concentration relationships are controlled by dilution (Walker 1987, 1990).

### Quality Assurance

Quantification of metals concentrations was based on calibration curves of standard solutions of the metals. Detection limits were: Cr: <0.07 µg/L; Cu: <0.01 µg/L; Zn: <0.1 µg/L; Pb: <0.06 µg/L. The precision of the analytical procedures expressed as the relative standard deviation (RSD) was 5–10%.The ICP-MS measurements always had an RSD of <5%. For all samplings, blanks were run and corrections applied, if necessary. All analyses were done in triplicate.

## Results

### Discharges of the Leyole and Worka rivers

**Fig. 3:**
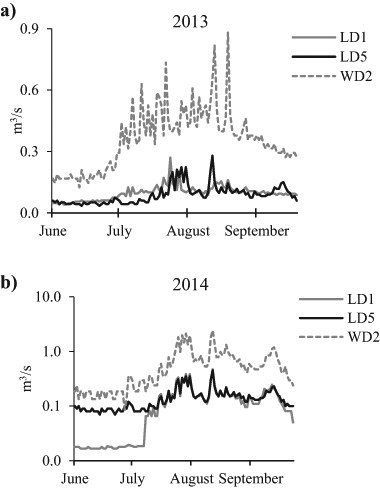
Water flows of the rivers. **a** Water flows (m^3^/s) of upstream Leyole river at station LD1, **b** downstream at station LD5, and **c** at the downstream Worka river station WD2, from 1 June to 30 September 2013 and 2014. Note the logarithmic scale in Fig. 3b

The hydrological flows of the Leyole and Worka rivers are modified by midstream industrial effluents and up-downstream agricultural activities along the rivers (Fig. 1). Though the rivers are having a width >4 m and depth of 3–5 m, the flowing water depth and width were not more than 1.25 and 2 m, respectively. For both 2013 and 2014 (Fig. 3), highest discharges were observed at all stations in July and August, as a result of increased rainfall, and reaching maximum discharge rates of approximately 0.9 and 1.3 m^3^/s in the Leyole and Worka rivers, respectively (Fig. 3). In the upstream part of the Leyole river (just downstream LD1; Fig. 2), daily mean flow rates ± standard error (*n* = 122) in the rainy seasons of 2013 and 2014 amounted to 0.12 ± 0.01 and 0.18 ± 0.11 m^3^/s, respectively. For the downstream part of the Leyole river, at LD5 (Fig. 2), these values were 0.14 ± 0.02 m^3^/s. and 0.28 ± 0.28 m^3^/s, respectively. Comparing the upstream and downstream flows, the dilution factors for the average flows in the downstream zone of the Leyole river amounted to 45% in the 2013 and 61% in the 2014 campaign. Similarly, based on campaign comparison, the dilution factor increased in 2014 by 88 to 108% upstream and downstream for the Leyole river, respectively. For downstream Worka river, at WD2 (Fig. 2), the mean daily flow rates were 0.36 ± 0.05 and 1.3 ± 0.1 m^3^/s, for the rainy season of 2013 and 2014, respectively (Fig. 3). The low river discharges reflect the area’s semi-arid climate.

### Metals in the Effluents and Effluents Mixing Zones of the River Waters

#### Metals in the effluents of the five factories

In the following, the 2013 campaign will be indicated as C1, the 2014 campaign as C2. In virtually all cases, the metal concentrations were distributed asymmetrically, with mean values affected by a few high or low values (Table 1). The EC for the steel processing effluent was found to be higher than for the other factories effluents (Table 1), though the high values of the standard errors make it hard to give definite conclusions. This effluent was also acidic, probably because of pickling acids used to remove oxides from steel surfaces. In contrast, the effluent from the brewery was alkaline, attaining pH values > 11, likely coming from detergents used for washing equipment.

Metal concentrations in the effluents were often characterized by high extremes and marked differences between mean and median values (Table 1). For the C2 campaign, Cr (median: 26,800 µg/L; maximum: *ca*. 65,000 µg/L) was very high in the tannery effluents compared with the other factories’ effluents. The relatively low Cr (median: 6.1 µg/L) contents in tannery effluents for campaign C1, and corresponding Cr loadings (Table 1), can be ascribed entirely to the cessation of the tanning processing during this first campaign. Cr median concentrations in the tannery factory effluents (Table 1) exceeded the guidelines values of both USEPA (US Environmental Protection Agency) and EMoI (Ethiopian Ministry of Industry).

In contrast to Cr, Cu effluent concentrations were below the two quality guideline values for all factories, but with noticeably higher concentrations in the steel processing factory than in the other effluents (Table 1). However, owing to larger effluent water discharges, higher Cu effluent loadings (g/day) were periodically observed for the tannery, brewery and textile factory. Zn effluent concentrations were particularly high in the steel factory effluents, for both campaigns, with higher median concentrations in 2014, when the steel galvanizing processing was expanded (Table 1). The Zn concentrations in the steel factory effluents far exceeded the USEPA and EMoI guidelines during the two campaigns (Table 1). The loading of Zn from the textile factory was also relatively high, though less marked, during the C1 campaign (no guidelines are set for Zn in textile and tannery effluents; Table 1). The mean Pb concentrations and loadings increased, largely from tannery effluents, during the C2-campaign (Table 1). Although, no guidelines are set for Pb in tannery effluents, maximum Pb values exceeded the guideline values set for Pb in the steel processing and textile industries effluents (Table 1). The expansion of the steel processing factory in 2014 may have resulted in increased Pb concentrations in the effluents during the C2-campaign.

#### Metals in the effluent mixing zones of the Leyole and Worka rivers

The upstream catchments of the Leyole and Worka rivers are largely under agricultural use and in the upper parts, stations LD1 and WD1 were considered as “background” stations to compare with the concentrations of metals downstream. However, at LD2, some increased Cr, Cu, and Zn concentrations were observed (Table 2). In contrast, at WD1, the median concentrations of all metals were lower than at WD2. EC values were somewhat higher in the effluents mixing zones for the tannery, meat processing and brewery factories, similar to the earlier mentioned EC values in their effluents (Tables 1, 2). In contrast, though EC was highest in the steel processing effluents, these effluents were largely diluted with river water and, therefore, no increased EC values in the steel factory’s mixing zone were observed compared with the other mixing zones. Similarly, no marked pH effects were observed in the effluent mixing zones, except for high pH values downstream of the brewery, in 2014 (Table 2).

The effect of the factories effluent discharges on the metal concentrations in the downstream river water was examined in the effluents mixing zones of the Leyole and Worka rivers (Fig. 2; Table 2). The Cr concentrations were highest at LD4 for the C2 campaign (median: 2660 µg Cr/L), similar to the tannery factory effluent itself (factory not operational during 2013 campaign; Table 1). The median Cr concentration at the tannery effluent mixing zone was increased by a factor 52 (2660 vs. 51 µg Cr/L) compared with the nearest upstream station for the C2 campaign (Table 2). Although Cr concentrations at LD5 were still relatively high, there was a marked decrease compared with LD4, probably due to increased dilution from numerous small streams flowing into the Leyole river between LD4 and LD5. In line with the observed effluent Cu concentrations (Table 1), no markedly increased Cu concentrations were observed in the mixing zones of the Leyole river except for relatively high median Cu concentrations during campaign C1 at LD3 in the textile effluent mixing zone (Table 2). In the Worka river, the median Cu concentration for both the C1- and C2-campaigns was higher at WD2, the mixing zone of the brewery effluent, than at WD1 (Table 2). No comparable increases at WD2 were observed for the other metals.

Consistent with Zn in the steel processing factory effluent (Table 1), highest Zn concentrations were found at the effluent mixing zone (LD2) with medians of 95 µg Zn/L and 521 µg Zn/L during C1 and C2, respectively. Just as for Cr, the Zn river concentration decreased again at LD3 (textile effluent mixing zone), reflecting a dilution effect from numerous water inflows into the river (Table 2). The median and mean of Pb concentrations in the effluent mixing zones for both the Leyole and Worka rivers were both quite low, comparable with Pb values at LD1.

Finally we tried to match, for both the Leyole and Worka rivers, the metal loadings (g/day) as calculated from the factories’ discharges (Table 1), with those calculated at the effluent mixing zones, as products of median metal river concentrations with river discharges (Table 2). Since the Leyole river discharges were not measured between LD1 and LD5, we assumed, by linear interpolation based on the distances between stations, that river discharges at LD2, 3 and 4 amounted to, respectively: 120, 135 and 138 L/s, for campaign C1, and 240, 277, and 287 L/s, for C2 (Tables 1, 2). Important results for these comparisons were, apart from extreme Zn and Cr loadings, rarely found (see later).

## Discussion

### Industrial Development and Pollution Management in the Kombolcha Industrial Zone

In 2010, the Ethiopian government implemented a 5 year Growth and Transformation Plan (GTP) through industrial growth and development. To realize industrial growth, the government identified five suitable sites (EMoI 2014). Here collaboration takes place with the International Development Association of the World Bank to implement the Industrial Development Zones Projects (IDZPs). The GTP is currently in the second (GTP II) of three phases in the planned transition as national structural changes from an agriculturally to industrially-led economy. After the structural changes have been effected, the government envisages, in the third phase (GTP III), to attain a middle- income state (per-capita income of 1200 USD per year) by the end of 2025.

Kombolcha, one of the five national IDZPs sites, is considered an ideal location because of its intermediate location for domestic markets exports via the Djibouti port (Fig. 1a). The city administration has allocated 1100 ha of land for industrialization (Mesfin 2012). Labor-intensive manufacturing industries are a priority area for the industrialization process. Abundant cheap labor force and opportunity for duty-free exports to the USA has stimulated international investors to engage in medium to large-scale manufacturing industries. Existing factories are also expanding. The BGI-brewery, and the Kombolcha textile and steel processing factories have recently undertaken major expansions. The Ethiopian Industrial Development Zone Corporation (EIDZC) is responsible for planning, implementation and supervision of environmental issues for the industrial projects. The regional and city environmental institutions are charged to ensure good environmental management of the projects. For the Kombolcha IZDP, the Amhara Regional Environmental Authority is responsible for coordinating the industrial pollution regulations. At local level, the Kombolcha Bureau of Environmental Protection, Land Administration and Use (EPLAU) is responsible for monitoring industrial pollution and evaluating compliance with environmental requirements.

The five factories examined in this study are located close to each other, with the new industries constructed in nearby areas. This will obviously increase pollution risks into receiving rivers. However, up until now we found no report dealing with environmental considerations for the Kombolcha IDZPs implementation, nor assessment studies on the carrying capacity of the surrounding environment with respect to expected industrial pollution.

### Industrial Effluents and Metals Pollution in the Kombolcha Industrial Zone

In the Kombolcha industrial zone, effluents discharged by each factory are managed independently. In spite of the close proximity of the factories, we observed no joint efforts by the factories to manage waste disposal. Currently no treatment facilities are present for the brewery (Table 3). For the other four industries, treatment takes place in lagoons or retaining ponds, but these facilities are quite old and designed to treat organic and sediment wastes only, rather than metal pollutants. According to the Environmental Pollution Control Proclamation of Ethiopia, all factories in the Kombolcha industrial zone are required to comply with national effluent emission standards, as each factory falls in the category for which emission standards are developed. Governmental environmental protection institutions both at the federal and regional levels coordinate the inspection of emission from the factories (for details, see next section) (FDRE 2002a; Afework et al. 2010; EEPA 2010).

**Table 3:** Expected effluent compositions for the five Kombolcha industries, type of treatment facility, and emission monitoring, as observed in 2015

Factory	Expected effluent composition	Treatment facility
Steel processing	toxics: As, CN, Cr, Cd, Cu, Fe, Hg, Pb, Zn; non-toxic: Fe^3+^, Ca^2+^, Mg^2+^, Mn^2+^.	Retaining ponds
Textile	Acid and alkaline, disinfectants: C1_2,_ H_2_O_2_, formalin, phenol	Facultative lagoons
Tannery	Cr and organic wastes (i.e. Bio- oxidizables (BOD))	Anaerobic lagoons
Meat processing	Organic wastes, suspended solids, and BOD, nutrients (P, N)	Anaerobic lagoons
Brewery	organic wastes, suspended solids, BOD, nutrients (P, N)	No treatment facility

Our study in 2013 and 2014 could only take place in the rainy seasons, when the effluents encountered higher dilutions owing to increased flows of the rivers. In the dry seasons, reduced dilution will lead to more serious pollution. The chromium in the tannery effluents comes from the commonly used chromium salt Cr_2_ (SO_4_)_3_12(H_2_O), for tannery processes (Pawlikowski et al. 2006; Akan et al. 2007). The low Cr concentration in the tannery effluents during the 2013 campaign (Table 1) can be attributed to the very low tanning production that year. According to the factory manager (Ali Mohammed, personal communication; 1 August, 2013), the factory process was then strictly limited to the preparatory steps before tanning, without the vegetal and chrome tanning processes involving Cr. In 2014, we found Cr concentrations as high as 64,600 µg/L in the tannery effluents exceeding both the USEPA and EMoI guidelines (Table 1). Similar observations are reported from other developing, and Sub-Saharan countries (Table 4). Though the dilution factors of the Leyole river increased during C2 compared with C1 (see section “*Discharges of the Leyole river and Worka rivers*”), Cr increased in the tannery effluent mixing zone, by a factor 51, compared with the nearest upstream station (Table 1) during full tannery production. With a comparable factory production capacity, Gebrekidan et al. (2009) and Katiyar (2011) reported enhanced river Cr concentrations downstream of the tannery effluent, at both high and low flows.

**Table 4:** Metals discharges from selected factories in Sub-Saharan and other developing countries

Factory effluent	Metals	Concentration (µg/L)	Country	Reference
Tannery	Cr	23,020	Kenya	Mwinyikione et al. (2006)
		10,820	Ethiopia	Gebrekidan et al. (2009)
		5790	Nigeria	Emmanuel and Adepeju (2015)
		3540	Ethiopia	Ayalew and Assefa (2014)
		264,000	Uganda	Oguttu et al. (2008)
		811,410	Morocco	Ilou et al. (2014)
		95,000	India	Ganesh et al. (2006)
		77,000	Albania	Floqi et al. (2007)
		5, 420,000	Bangladesh	Hashem et al. (2015)
	Pb	1060–1920	Nigeria	Akan et al. (2007)
		2870–3100	Nigeria	Emmanuel and Adepeju (2015)
		760	Morocco	Ilou et al. (2014)
		1970	Pakistan	Tariq et al. (2006)
Steel processing	Zn	5520	Nigeria	Adakole and Abolude (2009)
		2900	Bangladesh	Ahmed et al. (2012)
		168,150	Romania	Alexa (2013)
		498,500	India	Majumdar et al. (2007)
Textile	Cu	5140	Nigeria	Yusuff and Sonibare (2004)
		2200–4500	Nigeria	Ohioma et al. (2009)
		1090	Pakistan	Sial et al. (2006)
		1700	Pakistan	Manzoor et al. (2006)

In the effluents of the tannery factory, we also observed Pb peaks of up to 1670 µg/L when the factory was fully operational (Table 1). This is probably connected to the use of Pb in the finished and unfinished trim process in post tanning operation (Akan et al. 2007). For comparable cases of 15 tanneries in Pakistan (Tariq et al. 2006) and two in Nigeria (Akan et al. 2007), high Pb contents were observed as well, often leading to violation of water quality guidelines. Aklilu (2013) showed that Pb consistently exceeded FAO irrigation quality guidelines downstream of the tannery effluent mixing point of a river in Ethiopia.

The major operation of the steel processing factory is to heat and galvanize the steel products with zinc coats, leading to high Zn concentrations in the factory’s effluents (Tongpool et al. 2010). Zn in the Kombolcha factory effluents often exceeded both the USEPA and EMoI guidelines (Table 1). Similarly, very high Zn concentrations, up to 500 mg Zn/L have been recorded in effluents from steel processing factories elsewhere (Table 4). Tongpool et al. (2010) found that the hot dip-galvanized process resulted in major eco-toxicity. In our study, though the steel effluent was rich in Zn (Table 1), we found remarkably low Zn contents in the effluent mixing zone of the Leyole river (Table 2). This is due to the large dilution effect of the steel processing effluents into the Leyole river for the C1 and C2 campaigns, by factors of 70 and 109 (i.e. based on the effluent and river flows data (Tables 1, 2), respectively.

Cu was higher in the steel factory effluents and we also observed similar variations of Cu concentrations in the textile and steel effluent mixing zones (Table 2). Many studies reported high Cu concentrations in the effluents of textile factories, largely related to the coloring of the fabrics (Sial et al. 2006; Dwina et al. 2010; Ghaly et al. 2014). We hardly found elevated Cu concentrations in the textile effluents (Table 1), though the Cu concentrations were higher in the textile effluent mixing zone than for both the textile effluents and the other effluent mixing zones in the rivers (Table 2). In July 2014, a visit to the textile factory, indicated that treatment of effluent waste comprised only a facultative lagoon constructed to treat organic wastes rather than dissolved metals. Depending on the chemical products used for dyeing and the textile wet processing which is done at different times, pollutants in the effluent vary with time (Choudhury 2006) and thus, the monitoring interval for this study (i.e. 2 weeks) may also not have been adequate to capture the variations of Cu concentrations in the effluent. In general, the quantity and quality of industrial effluents vary with discharges, operation start-ups and shutdowns, and working hours distributions (Henze and Comeau 2008). While more frequent and preferably continuous sampling would have been desirable, this was not possible within the resource of the project.

Generally, we found similar trends for the metals concentrations in the factories’ effluents compared with those in the effluent mixing zones of the rivers (Tables 1, 2). Cr and Zn concentrations in the effluents of, especially, the tannery and steel processing factories provide clear evidence of pollution. Cu and Pb showed similar trends, though less frequent compared with Cr and Zn. The effluents from the brewery and the meat processing factories showed relatively low metal concentrations (Table 1). These effluents primarily comprise biodegradable/non-degradable organics and suspended solids, as well as nutrients such as ammonia, nitrate and phosphate (Inyang et al. 2012). More details are presented elsewhere (Zinabu et al. 2017).

For each metal, we found large differences in estimated loadings (g/day) from the effluents and the mixing zones (Tables 2, 3). Even though the frequency of monitoring the effluents and mixing zones were synchronized in this study, this was not always the case for the measurement of the effluent and river flows. For the Leyole river, the relative large influence of metal loadings from the upstream “background station” LD1 (Table 2) distort comparisons, but not the overall conclusions concerting high industrial driven pollution. The impact of this station, e.g. as a source of diffuse metal loadings, as well as effects of the metal loadings on the river and sediment qualities in the region, will be discussed elsewhere (Zinabu et al., in prep.).

The large differences between the effluent and river water discharges (for Leyole river by a factor of 9–109; Worka river: 44–63) (Tables 1, 2), with, at the same time, relatively low metal river water concentrations, was an additional factor for the large loading differences. Even for the extremely high Cr discharges from the tannery during campaign C2, there was a factor 3.6 (66,000/18,500) difference between the estimated effluent and stream loadings (Tables 1, 2). For the Zn discharges from the steel factory during campaign C2, relatively less difference was found with a factor of 1.6 (17,300/10,800) between the two estimated loadings (Tables 1, 2). Effluent impacts in the effluent mixing zone of receiving rivers is generally affected by variations in river flows and geomorphology of the river flows receiving the effluents, as well as effluent density and temperature differences between effluents and receiving water (Schnurbusch 2000; Alonso et al. 2016). These factors likely affected pollutant transport in the mixing zones in the Leyole and Worka rivers. Finally, since the rainfall distribution in the Kombolcha catchments is erratic and river bank erosion is evident over large parts of the river, mostly because of overgrazing and lack of erosion protection measures, both the earlier defined “Zone of initial dilution” (ZID) and chronic mixing zone (impact zone) likely vary over time and space. Thus, the 5 m long mixing zone selected for our study will not always have represented the actual mixing zones of the effluents.

In sub-Saharan countries, estimating pollutants loadings from factories using frequent monitoring over long duration may be difficult, both technically and cost-wise. Infrastructure for monitoring works are generally limited and water quality information is scant (Driscoll et al. 2003; Kamiya et al. 2008). Thus other, more economical methods giving comparable results must be chosen. Relating factories’ specific metals loadings (i.e. emission factors) to the associated activities resulting into the metal discharges, may be more appropriate than estimating loadings based on frequent monitoring of pollutant concentrations and measurement of flows in rivers (USEPA 2014). This holds especially in case of easier and less-cost activities, and is more useful in areas where monitoring infrastructures are challenging and water sampling is problematic due to low hydrological flows, like the Leyole and Worka rivers (Fig. 3).

It is important to note that high metal concentrations in the upstream parts of both the Leyole and Worka rivers showed the presence of sources of metals other than the factories listed in this study. The existing landfills and intensive agricultural activities in the area are likely sources of these metals, and additional study is needed to assess their inputs. Comparing the metal loadings at LD1 and LD2 (Table 2), we estimated that, on average, the former contributed 47% to the latter loadings, with minimum and maximum values of 2% (Cu; campaign C2) and >100% (Cu; campaign C1), respectively.

### Industrial Pollution Control Policy and Implementation in Ethiopia

The Ethiopian Federal government has already formulated a series of environmental proclamations pertinent to sustainable development, including the proclamation of the Environmental protection organs (FDRE 2002a), the Environmental pollution control proclamation (FDRE 2002b), the Environmental Impact Assessment (EIA) proclamations policy (FDRE 2002c) and the Water resources and management proclamation (EMoWR 2000). Empowered by the Environmental pollution control Proclamation No. 300/2002, the EEPA (Ethiopian Environmental Protection Authority) has formulated practicable emission standards that are generally required to be fulfilled by eight categories of factories liable to it (EEPA 2010), but there are several weaknesses to the Ethiopian regulatory structure for pollution control (Table 5). The factories are responsible not to exceed emission standards and to dispose effluents in an environmentally sound manner (Article 4 (1)). A factory that discharges a potentially dangerous pollutant is required to immediately notify the competent environmental authority (Article 4(4)). Penalty for violating the regulations are referred as criminal code that is elaborated with Clauses (Article 14 and Part Five (Offences and Penalties, Articles 12 to 17)). EEPA is also in charge of supporting technical guidance for Environmental Institutions at regional and sectorial levels; the regional states in turn transfer tasks to local levels. Subsequently, EEPA has now evolved into the *Ministry of Environment, Forest and Climate change*, but it is not clear yet whether the tasks will be changed or not. Here, we assume that EEPA will only be promoted administratively to ministerial level and that the tasks will remain unchanged.

**Table 5:** Description of Ethiopian pollution regulation and control components and, analysis of strengths, weakness and and possible solutions

Issue	Industrial effluent pollution			
Pollution regulation and control				
Regulatory structures	Federal level (EEPA), Regional level (REPA), Local level (Kombolcha Bureau of Environmental Protection, Land Administration and Use (EPLAU))			
Regulatory organs	Federal environmental institutions and the Council (Ethiopian Ministry of Environment, Forest and Climate change), Regional environmental institutions, Sectorial environmental institutions			
Control and command	Emission standards (limits of effluent quality discharge into water for eight categories of industries includinga (EEPA 2010)):			
	Tanning and the production of leather goods; The manufacture of textiles; Extraction of mineral ores, the production of metals and metal products; The manufacture of cement and cement products; Preservation of woods and manufacture of wood products including furniture; The production of pulp, paper and paper products and; The manufacture and formulation of chemical products including pesticides.			
Strengths	Manifestation of Ethiopian Environmental Policy			
	Formulation of laws and regulation to control industrial pollution (proclamations of the environmental protection organs; Environmental Pollution Control proclamation; the Environmental Impact Assessment (EIA) proclamations; and the Water Resources and Management proclamation)			
Weaknesses	Priority given to development over environmental protection	Lack of regulatory oversight relating to EIA	Reliance on use of effluent limits	Absence of any requirement to monitor or report for compliance of effluent limits
Source of weaknesses	• Lack of awareness and political commitments to environmental protection• Absence of clear links between development objectives and environmental protection• Foreign investor indifference to environmental protection	• Lack of effective rules and legal enforcement for EIA• Lack of environmental protection awareness by EIA licensing bodies• Absence of political commitment• Lack of communication among EIA regulatory institutions	• Lack of financial and technical resources by concerned institutions• Lack of economic incentive• Limited monitoring infrastructure for effluent receiving environments such as rivers• Lack of clear protection guidelines to effluent mixing environments	• Absence of rules for clear monitoring schemes for industrial pollutants• Limited professional, technical/finance capacity• Absence of technology standards to control pollution by industries• Lack of enforcement to compliance emission guidelines• Lack of transparency (for public use) in monitoring records
Possible solutions	• Awareness raising of decision makers in environmental protection• Prioritizing sustainable development in policy formulation and guidance	• Reformulating clear rules and strict implementation of EIA legal enforcement• Systematic use of EIA and coordinating the tasks of EIA regulatory institutions (e.g. licensing organization and EEPA)	• Introducing economic incentives schemes i.e. collecting revenue from emission fees, taxes and subsidies• Expanding monitoring infrastructures• Developing effect based water quality guidelines after mixing of effluents in receiving water bodies	• Formulating clear rules for emission monitoring in industries• Developing technology based emission guidelines• Capacity building of emission controlling institutions• Strict follow up of legal enforcements• Public disclosure of available monitoring records• Development of environmental management systems linked with monitoring and reporting

The emission standards set by EEPA are only focused on a limited number of pollutants. In principle, the EEPA guidelines could be technology-based (i.e. best available techniques (BAT)) or environment-based (i.e. environmental quality objectives or standards (EQOs)). Both methods generally demand detailed technological, economic and environmental considerations (OECD 1999). Looking at the guidelines classification scheme based on eight industrial categories in Ethiopia (Table 5), it is clear that EEPA uses BAT permits as precautionary measures. As there are no guidelines after effluent mixing, it is impossible to clearly understand impacts of effluent emissions into receiving waters (Table 5).

To evaluate the Kombolcha industrial effluents, we used the more frequently updated USEPA guidelines. In 2014, the Ethiopian Ministry of Industry used these to prepare a draft Environmental guidelines framework financed by the World Bank (EMoI 2014). At the moment, these EMoI guidelines are only intended to be used for the specific conditions of two industrial zones in the capital city, Addis Ababa. The guidelines include emission limits for pollutants from the same eight industrial categories, but are more recent than the more general EEPA guidelines (Table 5).

According to the Environmental pollution control proclamation, both the federal and regional environmental protection authorities coordinate inspection of pollution sources to control violation (FDRE 2002c). The regional state is also authorized to adopt emission permits and to control the more stringent industrial pollution areas. However, many studies indicate that downstream rivers are heavily polluted because of industrial wastes (Beyene et al. 2009; Prabu 2009). While industrialization has been growing fast for the past two decades, capacity within the regions and local environmental institutions have not kept pace with effective implementation of policy measures (World-Bank 2015).

Since 2008, EEPA has issued Directives to prevent environmental pollution. For licensing investment, EIA has been mandatory since 2003, but has been poorly implemented (Demeke and Aklilu 2008; CEPG 2012). Manufacturing industries are required to implement an environmental management plan and undertake environmental audit. However, new factories are often approved by licensing institutions (such as Ministry of Trade and Industry and Ministry of Mines and Energy) that regularly lack expertise, without the consent of EEPA and Regional Environmental Protection Authorities (REPA). Rather than carrying out EIA before the start of a project, in close communication with EEPA, licensing institutions often seem to rely on probable project outcomes with respect to monitoring and enforcement (Table 5). An example is outlined by Getu (2009) and CEPG (2012) reported on several licensed floriculture industries severely polluting downstream aquatic resources by fertilizers and pesticides. In a similar case, Demeke and Aklilu (2008) pointed out how a foreign company was licensed, with no prior EIA, to work on biofuel projects on land located inside a wildlife sanctuary. In all cases, communication between the licensing institutions and EEPA/REPA was poor with failure to carry out EIA in a coordinated manner. EEPA has already formulated guidelines for environmental impact study reports in the eight industrial categories (Table 5, EEPA 2010), but the EIA proclamation lacks clear understanding on the legal liability for improper implementation among the licensing institutions, environmental councils and sector bodies. EIA is often seen as a hindrance to development (Demeke and Aklilu 2008; CEPG 2012). Similarly, Ruffeis et al. (2010) indicated that in Ethiopia the investment proclamation tends to prevail over the EIA proclamation; allowing licenses without any obligations for an EIA.

In Ethiopia, given the limited financial capacity of, especially domestic, investors, financing Cleaner Production and waste treatment facilities are a high burden (Getu 2009; EEPA 2010; CEPG 2012) and financial initiatives from government to support such investments are limited (Assefa 2008). In developing countries, where factories are often traditional and small-scale (Jining and Yi 2009), the rate of changing old technologies and adoption of environmentally sound ones is slow (Bertinelli et al. 2012; Rudi et al. 2012). The implementation of the Ethiopian government industrialization plan, which stimulates growth of industries in specific zones throughout the country, would benefit from strong regulatory structures and pollutant monitoring. On the other hand, a frequent lack of respect by foreign investors towards multilateral environmental agreements (MEAs) and national environmental laws is a problem in many sub-Saharan countries (OECD 2007). In our study area, the French Castel Group Company in Ethiopia, having a high awareness of the need for environmental protection as evinced from the Castel website (http://www.groupe-castel.com/en/environment/), has been operating for a number of years without effluent treatment facilities. During the study time the brewery indicated that a treatment system was pending, though it appears not yet to have been installed, nor was access to the factory allowed up the submission of this article. Finally, the public awareness on environmental protection are increasing in the Kombolcha, as evinced elsewhere in the sub-Saharan countries (Getu 2009; Prabu 2009; CEPG 2012). In our study, at the downstream of Worka river, farmers claimed that there has been declining crop production over the years because of the use of the brewery effluent mixed water for irrigation. Though the public can prosecute polluting industries violating environmental emission limits, it will be difficult to prove since pollution records, if kept at all, are stored centrally at the Federal and Regional environmental Institutions and hardly available for examination (Table 5). As indicated elsewhere in Ethiopia, public participation in EIA remains very limited in Kombolcha (Damtie and Bayou 2008).

## Conclusion

Over the years, the Kombolcha industrial zone has become attractive for domestic and foreign investors in, especially, manufacturing industries. The expansions of the existing and building of new industries has led to gradual pollutants increments and exemplify the challenges of industrial cities in sub-Saharan country’s cities. Metals, especially Cr in tannery and Zn in steel processing factory effluents, were exceeding effluent emission quality guidelines. For Kombolcha, we suggest studies on the carrying capacity of the rivers that receive these industrial effluents. Further, a single centralized waste treatment facility used by multiple industries could be an efficient and cost-effective initiative.

Though legislation on industrial emission permits, control and fines do exist, the capacity of the local and regional environmental protection institutions for industrial pollution strategies is very limited. The discrepancies on the institutional levels and disagreements between the environmental and investment policies and proclamations hampers successful enforcement of environmental pollution control. The non-adequate respect for (inter)national environmental agreements by (foreign) investors and the absence of governmental initiatives to support adoption of cleaner production techniques are other factors of importance. This study generally shows that the industrial investment path followed in the Kombolcha industrial zone is unsustainable with respect to environmental concerns for the rivers that receive the effluents.

To ensure effective implementation of environmental pollution control policies, the Ethiopian Federal and Regional governments could better facilitate local environmental controlling institutions with the required instrumentations and mechanisms for law enforcement. Investment and capacity building within local government’s agencies can then provide long-term development of procedures and environmental protection. Ultimately, environmental protection is a social choice, and mechanisms that better involve all stakeholders, from local public to international investors, provide for the necessary dialogue and support of environmental regulations for both the region’s and country’s long-term sustainable development.
